# Optic disc shape in patients with long-lasting unilateral esotropia and exotropia

**DOI:** 10.1186/s12886-019-1197-8

**Published:** 2019-08-16

**Authors:** Kunte Shang, Yi Dai, Hong Liu, Xiaomei Qu, Wen Wen, Jost B. Jonas

**Affiliations:** 1 Department of Ophthalmology & Visual Science, Eye & ENT Hospital, Shanghai Medical College, Fudan University, 83 Fenyang Road, Shanghai, 200031 China; 2 NHC Key Laboratory of Myopia, Key Laboratory of Myopia, Chinese Academy of Medical Sciences, Fudan University, Shanghai, 200031 China; 3 Shanghai Key Laboratory of Visual Impairment and Restoration, Fudan University, Shanghai, 200031 China; 4 Department of Ophthalmology, Medical Faculty Mannheim of the Ruprecht-Karls-University, Heidelberg, Germany

**Keywords:** Optic disc shape, Esotropia, Exotropia, Optic disc ovality, Optic nerve, Myopia

## Abstract

**Background:**

Horizontal eye movements have been proposed to induce biomechanical stress and strain on optic nerve head. Since strabismus may lead to sustained adduction or abduction, we investigate the effects of long lasting unilateral horizontal strabismus on the morphology of optic disc.

**Methods:**

The observational cross-sectional study included patients with unilateral constant horizontal strabismus lasting for more than two years. The patients underwent an ophthalmological examination including refraction and morphometry of the optic nerve head. A prism cover test using right angle glass prism was performed to measure the magnitude of the ocular deviation.

**Results:**

The study included 70 patients with a unilateral constant strabismus (35 esotropic patients, 35 exotropic patients) with a mean age of 26 ± 19 years, mean refractive error of − 0.72 ± 3.3 diopters, mean axial length of 23.8 ± 1.7 mm, and a mean angle of deviation of 87 ± 36 prism diopters (Chinese right-angle glass method) in the esotropic group and − 97 ± 29 prism diopters in the exotropic group. In the whole study population and taken separately in the esotropic group and exotropic group, the disc ovality index (defined as ratio of minimal-to-maximal optic disc diameter) did not differ significantly between the deviating eyes and the contralateral fixating eyes (all *P* > 0.05). As a corollary, the disc ovality index and the prevalence of parapapillary beta/gamma zone did not differ significantly between the esotropic group and the exotropic group (all *P* > 0.05).

**Conclusions:**

Optic disc ovality did not differ markedly among long-lasting esotropic eyes, exotropic eyes, and non-strabismic eyes. It suggests that optic disc shape may not be markedly influenced in non-highly myopic eyes by a potential backward pull of the optic nerve on the optic disc structures in adduction or abduction.

**Electronic supplementary material:**

The online version of this article (10.1186/s12886-019-1197-8) contains supplementary material, which is available to authorized users.

## Background

The shape of the optic nerve head can be described by the ratio of its minimal to maximal diameter (disc ovality index), and by its tilt/rotations around the vertical axis, the horizontal axis and the sagittal axis [1, 2]. The tilted disc is mostly from the vertical disc rotation with the disc nasal margin elevated relative to the temporal margin, while the disc ovality index has long been accepted to reflect such a tilted configuration [3, 4]. It has been described that a tilted disc originates from oblique insertion of the optic nerve at birth [5]. Recent findings, however, have revealed that tilted disc may also be an acquired feature that develops during myopic shift in childhood [6, 7].

The biomechanical effect of horizontal duction on optic nerve head and peripapillary structures is of great research interest recently by several groups. Models of infinite element analysis predicted that eye movements (in both adduction and abduction) could generate large deformations within the optic nerve head through the pulling action of the optic nerve sheaths. Such deformations could be as large as an intraocular pressure elevation to 50 mmHg [8, 9]. Magnetic resonance imaging (MRI) studies provided evidences that optic nerve straightening is present in adduction, suggesting tethering by the optic nerve sheath in adduction is a novel mechanical load on the globe [10, 11]. Deformation of the optic nerve head and peripapillary structures during horizontal duction has also been visualized using spectral-domain optical coherence tomography (OCT) [12, 13].

Hence, we conducted this study to address whether in horizontal strabismic eyes a backward pull by the optic nerve on the optic disc may prevail to explain the disc ovality and vertical disc rotation. For that purpose we included long lasting unilateral horizontal strabismic eyes and compared the optic disc shape between the deviating eyes and the contralateral fixating eyes. Due to geometric reasons, the optic nerve pull on the optic disc would be greater in esotropic eyes than in exotropic eyes [9–13].

## Methods

The hospital-based clinical observational study included patients who consecutively attended the hospital from May 1, 2017 to May 30, 2018, who had a constant esotropia or exotropia of at least 30-prism diopters at distance and near as measured by the prism cover test, and who underwent fundus photography. The study protocol and data collection adhered to the tenets of the Declaration of Helsinki and were approved by the Human Subjects Review Board of Eye, Ear, Nose and Throat Hospital of the Fudan University in Shanghai, China. Written informed consent was obtained from all participants (or their parent in the case of children under 16).

Inclusion criterion for inclusion into the study was the presence of a unilateral constant strabismus for at least 2 years. Individuals with alternating or intermittent strabismus or with a history of an ocular trauma, ophthalmic surgery or diseases other than strabismus were excluded. All study participants underwent a comprehensive ophthalmologic examination including assessment of best corrected visual acuity and refraction, examination of strabismus, slit-lamp based biomicroscopy of the anterior and posterior segment of the eye, photography of the optic nerve head (CR-DGI; Canon, Inc., Tokyo, Japan) and ocular biometry with determination of the axial length (Lenstar LS 900; Haag-Streit Co., Koeniz, Switzerland). Authors had access to information that could identify individual participants during data collection.

The ocular alignment was assessed using the Hirschberg light reflex test and the cover–uncover test. The cover test was conducted at the far distance (6 m) and at the near distance (33 cm). An additional measurement of the ocular alignment in the near was carried out one hour after a monocular occlusion of the deviating eye. The ocular dominance was determined with the alternative cover test. The invariably fixating eye was regarded as the dominant eye. Ocular movements (versions and ductions) were examined in the nine diagnostic directions of gaze with the head in the primary position. A prism cover test using right angle glass prism was performed to measure the magnitude of the ocular deviation. Of note, the ocular deviation measured with different shaped prism is different [14, 15]. The prisms commonly used in China are right angle glass prisms. While isosceles acrylic prisms are commonly used in European and American countries. It has been reported that 90 prism diopters measured with right angle glass prisms approximately equal to 60 prism diopters measured with isosceles acrylic prisms [16].

The shape (ovality index) of the optic disc was measured based on the optic disc photos. The ovality index was calculated as the ratio of the minimum diameter to the maximum diameter of the optic disc. According to the fundus photographs, we assessed the presence of the parapapillary beta/gamma zone at the temporal disc border. Beta/gamma zone was defined as the whitish zone upon ophthalmoscopy in the parapapillary region [5, 7]. Spectral-domain OCT (Spectralis, Heidelberg Engineering, Heidelberg, Germany) was performed if the peripapillary atrophy was difficult to be outlined on the fundus photographs. The assessment of the optic disc ovality index and the parapapillary beta/gamma zone were performed by two experienced examiners independently of each other (Additional file 1).

The statistical analysis was performed using SPSS software 25.0 (IBM-SPSS, Inc., Chicago, IL, USA). We calculated the means, standard deviations, medians and ranges of the main outcome parameters. The statistical significance of differences in the main outcome parameters between the deviating eye and the fixating eye in both, the esotropic group and the exotropic group, was analyzed by the paired sample t-test. Associations between the main outcome parameters in the whole study group were analyzed by the Spearman correlation coefficient. All *P*-values were two-sided and the statistical significance level was set at 0.05.

## Results

Seventy patients with a horizontal unilateral strabismus were included into the study, with 35 patients in the esotropic group and 35 patients in the exotropic group. The mean age was 26 ± 19 years (median: 21 years; range: 5–78 years). The mean duration of strabismus was 14 ± 15 years (median: 7 years; range: 2–68 years). The mean refractive error (spherical equivalent) was − 0.72 ± 3.3 diopters (median: 0 diopters; range: − 9.25 to + 6.75 diopters). The mean axial length was 23.8 ± 1.7 mm (median: 23.6 mm; range: 21.2–27.9 mm). The mean angle of deviation was 87 ± 36 prism diopters (Chinese right-angle glass method) in esotropic group and − 97 ± 29 prism diopters in exotropic group.

**Fig. 1:**
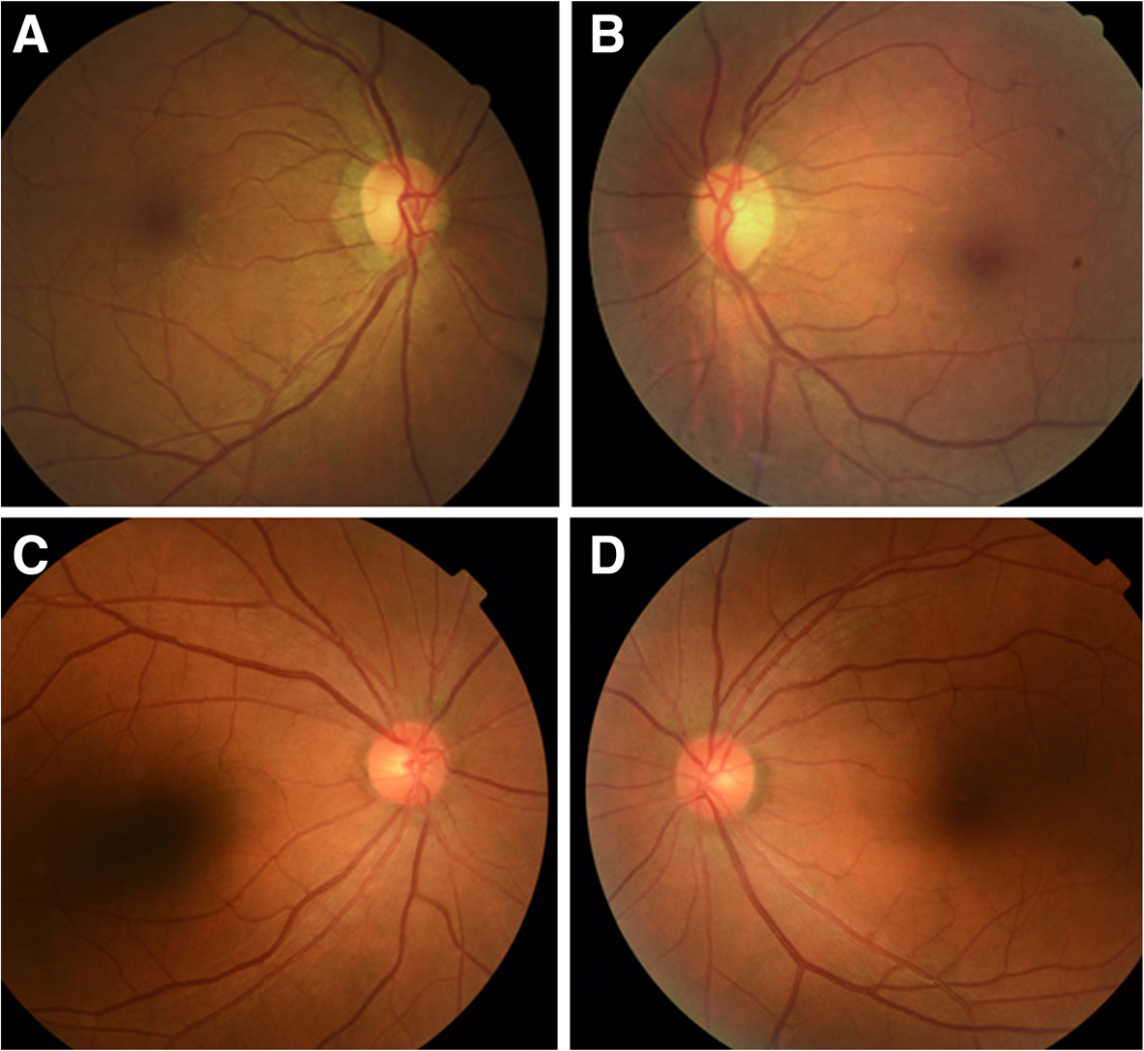
Representative fundus images of long-lasting unilateral esotropia. A female with the presence of a unilateral constant esotropia in right eye for about 52 years. Her axial length was 23.88 mm in right eye and 24.01 mm in left eye (**a**, **b**). A female with the presence of a unilateral constant esotropia in right eye for about 46 years. Her axial length was 23.02 mm in right eye and 23.04 mm in left eye (**c**, **d**)

In the whole study population, the optic disc ovality index did not differ significantly between the deviating eyes and the contralateral fixating eyes. The same held true when the analysis was performed separately in the esotropic group and in the exotropic group (all *P* > 0.05) (Table 1, Fig. 1). For esotropic group and exotropic group respectively, the refractive error, axial length and presence of parapapillary beta/gamma zone did not differ significantly between the deviating eye and the fixating eye in each groups (*P* > 0.05) (Table 1). We further excluded patients with anisometropia no less than 2D (*n* = 6 in the esotropic group, *n* = 2 in the exotropic group), the main ocular parameters between the deviating eye and the fixating eye still show no significant difference (*P* > 0.05).

**Table 1:** Main ocular parameters between the deviating and the contralateral fixating eyes

Variables	Exotropic group (*n* = 35)			Esotropic group (n = 35)		
	Deviating eye	Fixating eye	*P-*value	Deviating eye	Fixating eye	*P*-value
Refractive error (diopters)	−1.7 ± 2.7	−1.3 ± 2.5	0.066	0.11 ± 3.9	0.05 ± 3.8	0.804
Axial length (mm)	24.0 ± 1.7	23.8 ± 1.4	0.242	23.7 ± 1.9	23.7 ± 1.9	0.998
Optic disc ovality index	0.85 ± 0.09	0.86 ± 0.07	0.589	0.88 ± 0.07	0.87 ± 0.08	0.646
Presence of parapapillary beta/gamma zone (n (%))	22 (62.9)	15 (42.9)	0.051	16 (44.4)	16 (44.4)	1.00

Comparing the esotropic group with the exotropic group, the optic disc ovality index and the prevalence of parapapillary beta/gamma zone did not differ significantly between the esotropic group and the exotropic group (*P* > 0.05), while the deviation angle and refractive error differed markedly between 2 groups (*P* < 0.01), (Table 2). We further analyzed the relationship among these ocular parameters in the whole study cohort. The results showed that a more myopic refractive error was associated with a lower optic disc ovality index (*P* = 0.045), and higher prevalence of beta/gamma zone (*P* < 0.001) in the whole study group (*P* = 0.002). No significant association has been observed between disc ovality index and age or duration of strabismus, respectively (*P* > 0.05).

**Table 2:** Main ocular parameters between exotropic and esotropic eyes

Variables	Eyes in the exotropic group (*n* = 70)	Eyes in the esotropic group (*n* = 70)	*P*-value
Age (year)	28 ± 19	23 ± 18	0.128
Refractive error (diopters)	−1.5 ± 2.5	0.08 ± 3.8	0.004
Axial length (mm)	23.9 ± 1.5	23.7 ± 1.8	0.481
Deviation angle measured at a distance of 33 cm (prism diopter)	−97 ± 29	87 ± 36	< 0.001
Optic disc ovality index	0.86 ± 0.08	0.87 ± 0.08	0.218
Presence of beta/gamma zone (n (%))	46 (65.7)	32 (44.4)	0.32

## Discussion

In this observational study on patients with a unilateral constant strabismus, the optic disc ovality index did not differ significantly between the deviating eye and the contralateral fixating eye within an esotropic group and within an exotropic group nor between the exotropic eyes and the esotropic eyes within the total study population. Due to geometrical reasons with the optic nerve originating in the nasal upper region of the orbit, the potential backward pull of the optic nerve on the optic disc structures is more marked in adduction than in abduction, esotropic eyes as compared to exotropic eyes should have a more marked optic disc rotation around the vertical axis, if indeed the optic nerve exerts a backward pull by in these eyes. The lack of an association among the type of strabismus, the optic disc ovality (as index for vertical disc rotation) and presence of parapapillary beta/gamma zone suggests that in non-highly myopic eyes, the optic nerve may not exert a major backward pull.

The results of current study contrast with the previous findings that horizontal duction significantly deforms the optic nerve head and peripapillary tissues. Spectral-domain OCT studies showed that adduction displaced the nasal peripapillary Bruch’s membrane anteriorly and the temporal nasal peripapillary Bruch’s membrane posteriorly, leading to a tilting the optic nerve head around the vertical axis, with a reversal of this pattern in abduction [12, 13]. Studies using MRI revealed that the backward pull of the optic nerve on the optic nerve head in markedly axially elongated eyes could lead to a novel mechanical load on the globe at the optic nerve head in ocular adduction [10, 11]. It could explain the marked rotation of the optic nerve head around its vertical axis in highly elongated eyes, with the temporal border of the optic disc turning backward. The optic nerve traction on the temporal disc border could lead to an elongation of the peripapillary scleral flange and indirectly to the development and enlargement of parapapillary gamma zone [9, 17]. The reason for the discrepancy between the findings obtained in these previous studies and the observation made in our investigation may be the difference in axial length. Due to geometrical reason, the optic nerve is more likely to be tautened in adduction the longer the axial length of the eye is. One may assume that in non-highly myopic eyes, the length of the orbital part of the optic nerve is sufficient to allow the optic nerve to completely follow the movements of the eye, even in adduction.

If it is not the backward pull of the optic nerve leading to a vertical optic disc rotation in moderately myopic eyes, the question arises which other factors could be involved in the change of the optic disc shape during adolescence. Vertical disc rotation is most marked in myopic eyes, and that the amount of the vertical disc rotation is strongly correlated with the development and enlargement of parapapillary gamma zone [17]. The latter has been defined as the parapapillary region free of Bruch’s membrane and which is located in particular on the temporal side of the optic disc [18, 19]. Both, the vertical disc rotation and the development and enlargement of gamma zone were strongly associated with axial length and axial elongation. The studies by Kim and colleagues and by others demonstrated the marked rotation of the optic disc around the vertical axis in association with the development and enlargement of the parapapillary gamma zone in young individuals with progressive myopic axial elongation [6, 7].

The optic nerve head as a foramen in the posterior segment of the eye is anatomically composed of three layers. The superficial layer is the physiologic opening in Bruch’s membrane. The following layer is the foramen in the choroid, which is separated from the intrapapillary compartment by the peripapillary border tissue of Jacoby. The third and deepest layer is the foramen located in the level of the sclera and which is covered by the fenestrated lamina cribrosa. The latter is separated from the peripapillary scleral flange by the peripapillary border tissue of Elschnig [20]. At birth, all three layers are aligned to each other. With the growth of the eye, in particular during the process of emmetropization after the end of the second year of life, Bruch’s membrane opening may be moved in direction to the fovea due to a potential new production of Bruch’s membrane in the mid-periphery of the axially elongating eye [21]. Since the choroidal foramen within its optic disc hole and in particular the scleral foramen with the lamina cribrosa may not completely follow the temporal shift of the Bruch’s membrane opening, the optic nerve head may get an oblique course, with Bruch’s membrane opening being misaligned (in relationship to the choroidal foramen) in direction to the fovea, and the scleral foramen being misaligned (in relationship to the choroidal foramen) in direction to the nasal side. This phenomenon would explain the overhanging of Bruch’s membrane on the nasal side of the optic disc in moderately myopic eyes, and the development of parapapillary gamma zone as Bruch’s membrane free zone on the temporal side of the optic disc [22]. The phenomenon could also explain the paradoxical course of the optic nerve through the optic nerve head channel with the optic nerve entering the canal from nasal anteriorly and reaching the vitreous compartment temporal posteriorly, although the optic nerve arrives at the eye globe coming from the posterior part of the orbit. The described mechanism of a backward movement of Bruch’s membrane opening leading to its misalignment with the scleral foramen within the optic nerve head would not require the backward traction force by the optic nerve to change the ophthalmoscopical appearance of the optic disc from an almost circular structure to a vertically ovally configurated structure. The observation made in the present study supports such a notion since the optic disc ovality index did not differ between esotropic eyes and exotropic eyes.

Limitations of our study should be discussed. First, it was a hospital-based study with the possibility of a referral and selection bias. Since the study participants were however consecutively included into the study, a selection bias appears to be unlikely. Second, the optic disc rotations were not measured by optical coherence tomography. However, previous studies have demonstrated that there was a significant correlation between the disc ovality index and vertical disc rotation [23, 24]. Third, we did not obtain magnetic resonance imaging tomograms to assess the relationship of the length of the orbital part of the optic nerve and the position of the optic nerve head of the strabismic eyes.

## Conclusions

In conclusion, the results suggest that the disc ovality index did not differ markedly among esotropic eyes, exotropic eyes, and non-strabismic eyes in a non-highly myopic group. It suggests that the disc shape and vertical disc rotation were not markedly influenced by a potential backward pull of the optic nerve on the optic disc structures in adduction or abduction.

## Additional file

Additional file 1: Parameters on exotropic and esotropic eyes in all participants. (XLS 80 kb)

## Data Availability

All datasets generated and/or analyzed during this study are included in this article and its supplementary information files.

## Abbreviations

MRI: Magnetic resonance imaging
OCT: Optical coherence tomography
